# NLRP3 Overexpression Associated With Poor Prognosis and Presented as an Effective Therapeutic Target in Osteosarcoma

**DOI:** 10.3389/fphar.2021.724923

**Published:** 2021-07-28

**Authors:** Zhen Huang, Hui Chen, Shenglin Wang, Hongxiang Wei, Xinwen Wang, Rongkai Shen, Yunqing Wang, Rongjin Lin, Jianhua Lin

**Affiliations:** ^1^ Department of Rehabilitation, The First Affiliated Hospital of Fujian Medical University, Fuzhou, China; ^2^ Department of Orthopedics, The First Affiliated Hospital of Fujian Medical University, Fuzhou, China; ^3^ Fujian Orthopedics Research Institution, The First Affiliated Hospital of Fujian Medical University, Fuzhou, China; ^4^ Department of Nephrology, Shanghai East Hospital, School of Medicine, Tongji University, Shanghai, China; ^5^ Department of Orthopedics, The People’s Hospital of Jiangmen City, Southern Medical University, Jiangmen, China; ^6^ Department of Nursing, The First Affiliated Hospital of Fujian Medical University, Fuzhou, China

**Keywords:** osteosarcoma, prognosis, NLRP3, CY-09, lentivirus

## Abstract

Despite the development of diagnostic and treatment strategies, the survival outcome of patients with osteosarcoma remains poor. Nod-like receptor protein 3 (NLRP3) plays a crucial role in the inflammasome pathway, which is related to the progression of various tumors. However, the effect of NLRP3 on osteosarcoma has not yet been well explored. Our study aimed to investigate the role of NLRP3 in the malignant biological behavior of osteosarcoma as well as its therapeutic value. Immunohistochemistry was applied to investigate the NLRP3 expression in osteosarcoma and osteochondroma specimens. Cell Counting Kit-8, colony formation, wound healing, transwell, and flow cytometry assays were used to explore the contribution of NLRP3 to the proliferation, migration, invasion, apoptosis and cell cycle distribution of osteosarcoma cells *in vitro*. Western blot was performed to evaluate the expression of NLRP3 and the related proteins in osteosarcoma cell lines after the blockade of NLRP3 using CY-09 and lentivirus intervention. Furthermore, tumor formation assay was used to analyze the effect of NLRP3 on the growth of osteosarcoma *in vivo*. The results showed that the NLRP3 protein was overexpressed in osteosarcoma, which was independently correlated with the poor prognosis of patients. Moreover, NLRP3 suppression by the inhibitor of CY-09 or lentivirus-induced gene knockdown inhibited the cell proliferation, migration, invasion and promoted the cell apoptosis and G1 cell cycle arrest in osteosarcoma via targeting the inflammasome pathway. Our *in vivo* results confirmed that the inhibition of NLRP3 suppressed the tumor formation of osteosarcoma. In conclusion, NLRP3 may be regarded as an independent prognostic biomarker and a potential therapeutic target for osteosarcoma.

## Introduction

Osteosarcoma is the most common primary aggressive bone tumor that usually occurs during childhood and adolescence (Chow et al., 2020). With the development of novel therapies, the five-year survival rate of patients with osteosarcoma has improved from 20 to 70%. However, nearly 30% of osteosarcoma patients are prone to metastasis or recurrence, with an unfavorable prognosis, due to the limited efficacy of current treatment strategies (Dai et al., 2011). Therefore, exploring novel biomarkers and therapeutic targets for the treatment of osteosarcoma is critical.

Nod-like receptor protein 3 (NLRP3) has been intensively investigated for its potential role in various human diseases (Yang et al., 2019; Lin et al., 2020). The NLRP3 protein was also reported to play a vital role in activating and promoting of human cancers, including oral squamous cell carcinoma Wang et al. (2018), endometrial cancer Liu et al. (2019), and breast cancer (Li et al., 2020). According to Xue et al. (2019) patients with NLRP3 overexpression was found to have a poorer overall survival and disease-free survival five years after surgery in laryngeal squamous cell carcinoma, indicating NLRP3 serving as an auxiliary indicator for the long-term prognosis in patients with laryngeal squamous cell carcinoma. However, the prognostic and therapeutic values of NLRP3 protein have not been fully analyzed in osteosarcoma. Therefore, our study aimed to evaluate the expression of NLRP3 in osteosarcoma and its association with clinicopathological parameters of patients, and further explore the *in vitro* and *in vivo* effects of NLRP3 on the biological behaviors of osteosarcoma.

## Materials and Methods

### Tissue Samples and Patient Characteristics

In total, 55 patients with primary osteosarcoma and 30 patients with osteochondroma who have undergone surgical resection at our hospital were selected between January 1, 2012, and December 31, 2015. The clinicopathological parameters, including sex, age, tumor size, tumor location, histological subtype, Enneking stage, response to chemotherapy, and distant metastasis status, were recorded. The date of diagnosis to the date of first tumor progression was defined as progression-free survival (PFS), and the date of diagnosis to the date of death was defined as overall survival (OS). Patients were censored if tumor progression or death did not occur in the last follow-up. All patients participating in our study signed an informed consent form, and the study was conformed with the ethical guidelines of the Declaration of Helsinki.

### Immunohistochemistry (IHC)

NLRP3 protein expression in osteosarcoma and osteochondroma specimens was detected by immunohistochemical staining using a PV9000 immunohistochemical kit (OriGene Technologies, Inc., Beijing, China). Following deparaffinization, hydration, and blockage of endogenous peroxidase activity by 3% hydrogen peroxide, the specimen sections underwent antigen retrieval process in citrate buffer (pH 6.0) and were incubated overnight at 4°C with antibodies against NLRP3 (Affinity, United States) and Ki-67 (Affinity). Then, the sections were incubated with poly-peroxidase-anti-mouse/rabbit IgG and stained with diaminobenzidine (OriGene Technologies), followed by counterstaining with hematoxylin and dehydration. The results of IHC were evaluated by two pathologists independently. The grade of NLRP3 protein expression was scored from 0 to 3 according to the staining intensity of osteosarcoma cells (0, negative; 1, weakly positive; 2, moderately positive; 3, strongly positive). The mean percentage of positive osteosarcoma cells was also scored from 1 to 3 (1, <25%; 2, 25%–75%; 3, >75%) (Wang et al., 2018). The final scores were the grade multiplied by the percentage score of tumor cells, giving final scores of 0, 1, 2, 3, 4, 6, 9. Scores greater than 2 (3, 4, 6, 9) were considered as high expression, whereas those below or equal to 2 (0, 1, 2) were defined as low expression (Srirajaskanthan et al., 2010).

### Cell Culture

The human fetal osteoblastic cells hFOB 1.19 and human osteosarcoma cells MNNG/HOS and Saos2 were purchased from the Typical Culture Preservation Committee of the Chinese Academy of Sciences (Shanghai, China). 143B, MG63, and U2OS cell lines were obtained from the American Type Culture Collection (Manassas, VA, United States). The 143B, U2OS, MNNG/HOS and MG63 cells were cultured in Dulbecco’s modified Eagle’s medium (Biological Industries, Israel), and the Saos2 cells were was cultured in Roswell Park Memorial Institute 1,640 medium (Biological Industries). All medium were supplemented with 10% fetal bovine serum (FBS; Biological Industries) and 1% penicillin/streptomycin (Biological Industries) in an atmosphere of 5% CO_2_ at 34°C (hFOB 1.19) or 37°C (MNNG/HOS, MG63, 143B, U2OS and Saos2 cells).

### Lentivirus Induced NLRP3 Knockdown

Negative control vector sequences (sh-NC: TTC​TCC​GAA​CGT​GTC​ACG​T) and NLRP3 lentivirus sequences (sh-NLRP3: GTT​AGA​AAC​ACT​TCA​AGA​A) were obtained from GeneChem (Shanghai, China). Approximately 2 × 10^5^ cells/well were seeded into six-well plates. Then, the medium was replaced with 1 °ml fresh medium containing an appropriate amount of lentiviral suspension with an infection multiplicity of 50. After 24-h incubation, lentivirus-containing medium were replaced with fresh complete medium. The cells were incubated for another 48 h and green fluorescence was observed. Then, the cells were treated with puromycin for 2°weeks to screen stably transfected clones. The infection efficiency was further evaluated using western blot analysis.

### Western Blot Analysis

The cell proteins were extracted by using a Membrane and Cytosol Protein Extraction Kit (Beyotime Biotechnology, Shanghai, China). Then the proteins were then quantified using a Pierce^™^ Microplate BCA Protein Assay Kit (Thermo Fisher Scientific, Waltham, MA, United States). A total of 20 μg proteins were separated on a 10% sodium dodecyl sulfate-polyacrylamide gel and then transferred onto polyvinylidene difluoride membranes (Millipore, Bedford, MA, United States). All membranes were cultured overnight at 4°C with primary antibodies against NLRP3 (Affinity), caspase-1 (Affinity), gasdermin d (GSDMD, Affinity), IL-1β (Affinity), IL-18 (Affinity) and tubulin (Affinity) after blockage by 5% skim milk for 1 h. Then the membranes were incubated with appropriate secondary antibodies (Affinity) at room temperature for 1 h. Finally, the blots were detected by using an ECL Western Blotting Substrate (Affinity) and FluorChem R detection system (ProteinSimple, United States).

### Cell Viability Assays

Cell Counting Kit-8 (CCK-8; New Cell & Molecular Biotech, China) was used to measure the cell viability of osteosarcoma. Briefly, the 143B, MG63, MNNG/HOS, Saos2, and U2OS cell lines were seeded into 96-well plates at a density of 1.5 × 10^3^ cells/well and cultured at 37°C for 24°h, followed by incubation in a medium containing different concentrations of CY-09 for 24, 48, and 72 h. Cells with lentivirus infection were cultured at a density of 1.5 × 10^3^ cells/well on a 96-well plate for 24, 48, 72, and 96°h, followed by the supplement of 100 μL medium which containing 10 μL of CCK-8 solution to each well and then incubation at 37°C for 2 h. Absorbance (OD_450_) was detected at a wavelength of 450 nm using a microplate reader (ELx800; BioTek Instruments, United States). The experiment was carried out in triplicate.

### Colony Formation

Colony formation assay was applied to detect the effect of NLRP3 inhibition on cell proliferation. 143B and U2OS cells were seeded into six-well plates at a density of 200 cells/well and treated with different concentrations of CY-09 (0, 5, 10, and 20 μM). Simultaneously, 143B and U2OS cells in sh-NLRP3 and sh-NC groups were seeded into six-well plates at a density of 200 cells/well. After incubation for seven days, the cells were fixed with ice-cold methanol for 20 min and stained with crystal violet (Solarbio, Beijing, China). Colonies including more than 50 cells were counted using an optical microscope (Carl Zeiss, Germany). The experiment was carried out in triplicate.

### Wound Healing Assay

Both 143B and U2OS cells were incubated at a density of 5 × 10^5^ cells/well in six-well plates for 24 h. Then, a 200 μL sterile pipette was used to make scratches, and cells were then cultured with CY-09 at concentrations of 0, 5, 10, and 20 μM in FBS-free medium. Simultaneously, 143B and U2OS cells in sh-NLRP3 and sh-NC groups (5 × 10^5^ cells/well) were seeded into six-well plates and scratched with a 200 μL sterile pipette, and cultured in FBS-free medium. Images of wound healing were then acquired at the time of wounding and, 12, 24, and 36 h later. The experiment was carried out in triplicate.

### Transwell Assay

Transwell assay was conducted to explore the cell migration and invasion of osteosarcoma using chamber with 8-μm pores (Corning Inc., Corning, NY, United States). For invasion assay, the upper chamber was precoated with Matrigel solution for 2 h (BD Biosciences, Franklin Lakes, NJ, United States). Cells (1 × 10^5^ cells/well) pretreated with CY-09 or lentivirus infection were cultured in the upper chamber in 100 μl of FBS-free medium, with 600 μL of complete medium adding to the lower chamber in 24-well plates. After incubation for 24°h, the cells remaining in the upper side of chamber (non-migrating or non-invading cells) were removed. The migrated or invaded cells in the lower side were fixed with methanol for 20°min, stained with 0.1% crystal violet (Solarbio) for 30 min and counted under the inverted microscope. The experiment was performed in triplicate.

### Flow Cytometry

143B and U2OS cells were seeded with approximately 2 × 10^5^ cells/well into six-well plates and incubated with different concentrations of CY-09 (0, 5, 10 and 20 μM). sh-NLRP3 and sh-NC cells were cultured on six-well plates at the same density as before (2 × 10^5^ cells/well). After incubation for 48°h, a Cell Cycle and Apoptosis Analysis Kit (Beyotime Biotechnology) was applied to detect cell apoptosis and cell cycle changes in 143B and U2OS cells. Then the samples were assessed through a flow cytometer (BD Biosciences), and evaluated using the Accuri C6 software (BD Biosciences). The experiment was conducted in triplicate.

### Animal Experiments

The animal experiments were conducted according to the Committee of Animal Ethics of our hospital. Nude BALB/c mice (four to six weeks old) were purchased from the Charles River (Beijing, China). To investigate the effect of CY-09 on the tumor growth, 143B cells (2 × 10^6^/mouse) were injected into the left subcutaneous region of the mice. After the tumor volume reached 200 mm^3^, CY-09 (10 mg/kg) or DMSO was injected into the tail vein every day for the first 3°days and then every second day for 20 consecutive days. Tumor size was measured every week after administration. To investigate the effect of NLRP3 knockdown on the tumor growth, sh-NLRP3 and sh-NC cells (2 × 10^6^ cells/mouse) were injected into the left subcutaneous region of the mice. Tumor size was measured every week after transplantation. Then, the mice were sacrificed and tumor tissues underwent H&E staining, and Ki-67 immunohistochemical staining to examine the progression viability in different groups.

### Statistical Analysis

The statistical analysis was conducted using SPSS software version 19.0 (SPSS, Chicago, United States). Student’s *t*-test or One-way ANOVA was applied for two-group comparisons. The survival status was investigated using log-rank test and Kaplan-Meier survival plots. Cox proportional hazards regression was performed for multivariate analysis of parameters which were significant in univariate analysis. Proportional hazards assumption was validated via the log-minus-log-survival curves and survival times against cumulative survival using SPSS software version 19.0. The Harrell concordance index (C-index) was applied to evaluate the accuracy of the model. The optimal cutoff value of the NLRP3 expression scores for PFS and OS was determined by receiver operating characteristic (ROC) curve and Youden Index (Schisterman et al., 2005). *p*-values less than 0.05 were considered statistically significant.

## Results

### NLRP3 Expression in Osteosarcoma and Its Association With Patient Survival

**FIGURE 1:**
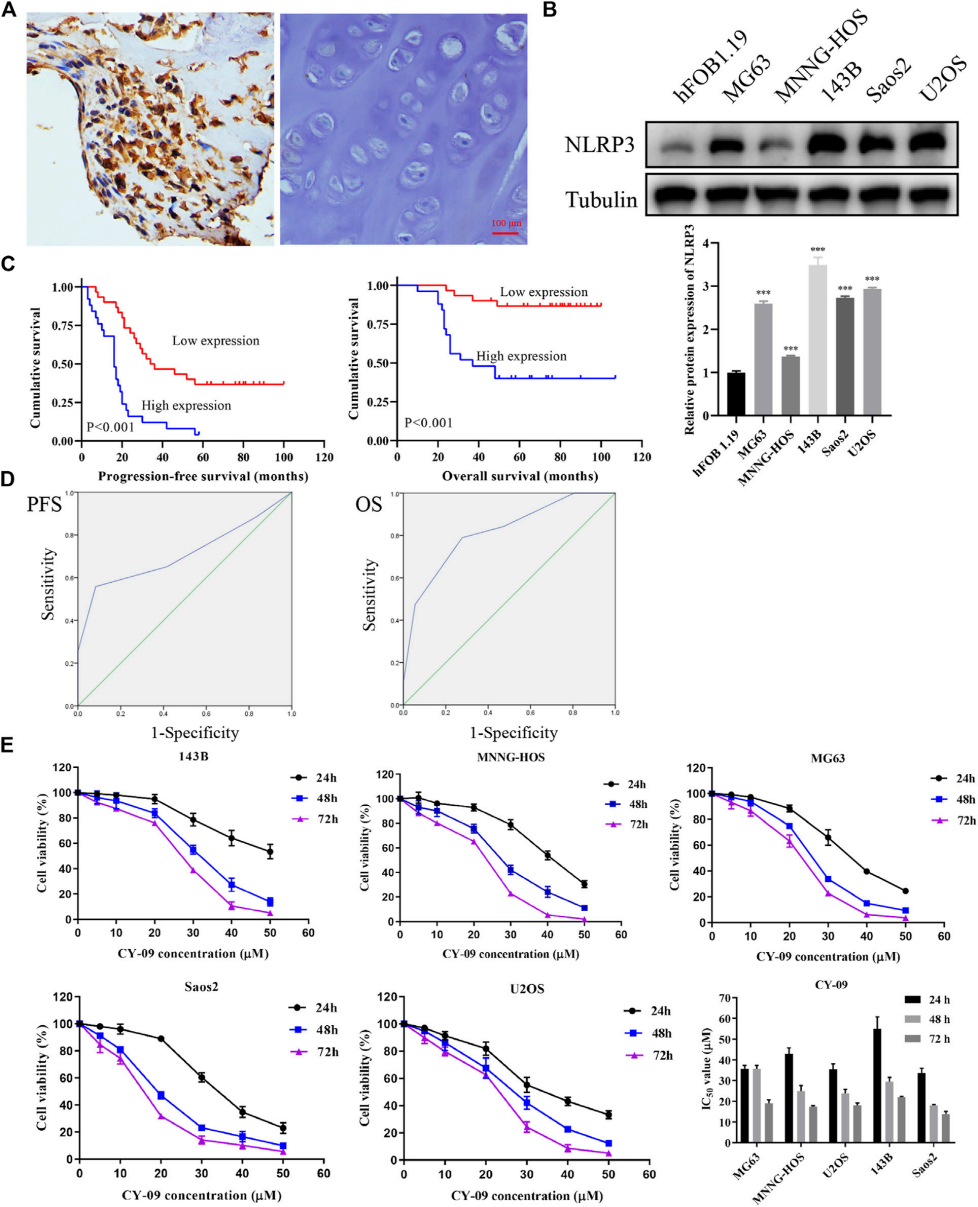
NLRP3 expression in osteosarcoma and cell viability changes after CY-09 administration **(A)** NLRP3 expression in osteosarcoma and osteochondroma tissues **(B)** NLRP3 expression in hFOB 1.19 and osteosarcoma cell lines **(C)** Prognostic value of NLRP3 for patients with osteosarcoma **(D)** ROC curves for PFS and OS in osteosarcoma with different expression scores of NLRP3 protein **(E)** Cell viability changes in osteosarcoma cells after treatment with different concentrations of CY-09. The NLRP3 expression in cells and the cell viability were measured in triplicate.

IHC was first used to investigate the expression of NLRP3 in 55 osteosarcoma and 30 osteochondroma tissues. In total, high expression of NLRP3 protein was found in 45.45% (25/55) of the osteosarcoma tissues whereas not in the osteochondroma tissues. The difference in the NLRP3 expression between osteosarcoma and osteochondroma specimens was significant (Figure 1A). The western blotting assay showed that NLRP3 protein was upregulated in MG63, MNNG/HOS, 143B, U2OS and Saos2 cells compared to the hFOB 1.19 cells, with 143B and U2OS cells exhibiting the highest level (Figure 1B). Furthermore, high expression of NLRP3 was significantly related to poor response to chemotherapy (Table 1, *p* = 0.021) and distant metastasis (Table 1, *p* = 0.013). Univariate and multivariate analyses were performed to assess the contribution of NLRP3 high expression to PFS and OS in osteosarcoma. According to univariate analysis, NLRP3 high expression (PFS, *p* < 0.001; OS, *p* < 0.001; Figure 1C), response to chemotherapy (PFS, *p* = 0.047; OS, *p* = 0.002), and distant metastasis (PFS, *p* = 0.002; OS, *p* = 0.003) were closely related to PFS and OS (Table 2). The multivariate analysis further confirmed the independent prognostic value of NLRP3 protein overexpression for PFS (hazard ratio (HR) = 2.92, *p* = 0.002, C-index = 0.70) and OS (HR = 4.09, *p* = 0.017, C-index = 0.79) in osteosarcoma patients (Table 3). The ROC curve and Youden index revealed that the optimal cutoff value of NLRP3 protein expression for PFS was 3.0 (area under the curve = 0.712, *p* = 0.026, sensitivity = 55.8%, specificity = 91.7%, Youden’s index = 0.475), and OS was 3.0 (area under the curve = 0.817, *p* < 0.001, sensitivity = 78.9%, specificity = 72.2%, Youden’s index = 0.515) (Figure 1D; Table 4). Our definition of NLRP3 expression level according to immunohistochemical scores was consistent with the optimal cutoff value for PFS and OS. Therefore, high expression of NLRP3 protein may be considered as an independent prognostic biomarker for osteosarcoma.

**TABLE 1:** Association between NLRP3 expression and clinicopathological characteristics in osteosarcoma.

Clinicopathologic data	Case number	High-expression (*n* = 25)	Low-expression (n = 30)	*p*-value
Sex				
Male	36	17	19	0.717
Female	19	8	11	
Age (years)				
<18	23	11	12	0.765
≥18	32	14	18	
Tumor size (cm)				
<8	32	16	16	0.425
≥8	23	9	14	
Tumor location				
Tibia and Femur	40	18	22	0.912
Others	15	7	8	
Histologic subtype				
Conventional	52	23	29	0.585
Special	3	2	1	
Enneking staging				
I-IIA	14	6	8	0.821
IIB	41	19	22	
Response to chemotherapy				
Good	28	17	11	0.021
Poor	27	8	19	
Distant metastasis				
Yes	23	15	8	0.013
No	32	10	22	

**TABLE 2:** Univariate analysis of NLRP3 expression and osteosarcoma patients survival.

Characteristics	Case number	Progression-free survival (months)				Overall survival (months)			
		Mean	SD	95%CI	*p*-value	Mean	SD	95%CI	*p*-value
Sex									
Male	36	34.53	4.73	25.27–43.79	0.583	80.65	6.27	68.33–92.97	0.818
Female	19	43.53	8.90	26.09–60.96		73.95	7.93	58.41–89.49	
Age (years)									
<18	23	34.00	5.62	22.98–45.02	0.702	68.57	7.13	54.58–82.55	0.285
≥18	32	40.25	6.373	27.76–52.74		84.39	6.45	71.74–97.04	
Tumor size (cm)									
<8	32	38.88	6.41	26.30–51.48	0.967	77.07	6.90	63.55–90.58	0.570
≥8	23	36.44	5.80	25.07–47.80		72.57	6.57	64.68–90.45	
Tumor location									
Tibia or femur	40	42.75	5.56	31.86–53.64	0.101	83.88	5.67	72.77–94.98	0.177
Other location	15	26.40	6.80	13.08–39.72		61.07	8.60	44.22–77.91	
Histologic subtype									
Conventional	52	36.15	4.22	27.88–44.42	0.694	79.96	5.21	69.74–90.18	0.902
Special	3	46.67	21.82	3.90–89.43		73.33	21.77	30.66–116.01	
Enneking staging									
I-IIA	14	33.07	8.28	16.84–49.30	0.668	72.21	10.93	50.79–93.64	0.369
IIB	41	39.93	5.35	29.45–50.40		77.72	5.16	67.60–87.84	
Response to chemotherapy									
Good	28	47.64	7.25	33.43–61.86	0.047	94.68	5.72	83.47–105.89	0.002
Poor	27	27.26	4.09	19.25–35.27		57.65	6.04	45.81–69.50	
Distant metastasis									
Yes	23	21.83	2.38	17.16–26.49	0.002	62.74	8.27	46.53–78.95	0.003
No	32	50.88	7.07	37.02–64.73		86.56	4.99	76.79–96.33	
NLRP3 expression									
High	25	18.92	2.82	13.40–24.44	<0.001	59.08	7.97	43.45–74.71	<0.001
Low	30	53.83	6.70	40.71–66.96		91.20	4.14	83.09–99.31	

**TABLE 3:** Multivariate analysis of NLRP3 expression and osteosarcoma patients survival.

Characteristics	Comparison	Progression-free survival (months)			Overall survival (months)		
		HR	95%CI	*p*-value	HR	95%CI	*p*-value
NLRP3	High vs low	2.92	1.40–6.05	0.002	4.09	1.29–12.97	0.017
Response to chemotherapy	Good vs poor	1.03	0.52–2.06	0.931	2.57	0.81–8.17	0.109
Distant metastasis	Yes vs no	1.88	1.00–3.71	0.068	2.31	0.85–6.28	0.100
C-index (95%CI)	—	0.70	0.61–0.78	—	0.79	0.70–0.89	—

**TABLE 4:** ROC curve analysis for PFS and OS in osteosarcoma with different expression scores of NLRP3 protein.

	AUROC	*p*-value	Cutoff value	Sensitivity%	Specificity%	Youden’s index
PFS	0.712	0.026	3.00	55.8	91.7	0.475
OS	0.817	<0.001	3.00	78.9	72.2	0.511

### NLRP3 Inhibition Suppresses Cell Proliferation, Migration and Invasion in Osteosarcoma

**FIGURE 2:**
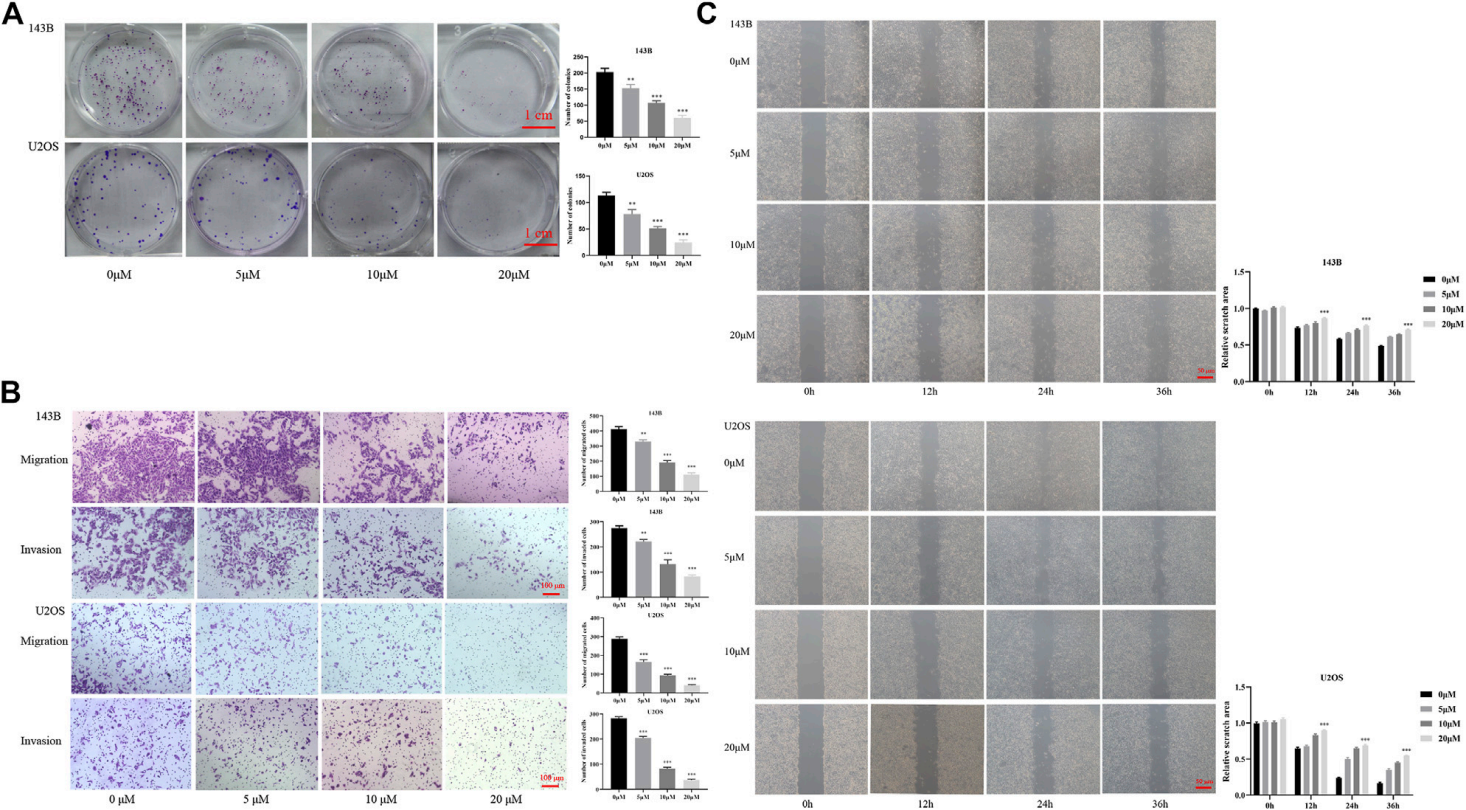
Effect of CY-09 on cell proliferation, migration, and invasion in osteosarcoma **(A)** Cell proliferation of 143B and U2OS cells detected by colony formation assay after treatment with different concentrations of CY-09 for seven days **(B)** Cell migration and invasion of 143B and U2OS cells were examined using the transwell assay after treatment with different concentrations of CY-09 **(C)** Cell migration of 143B and U2OS cells was evaluated using the wound healing assay after treatment with different concentrations of CY-09 for 0, 12, 24, and 36 h. The experiments were conducted in triplicate.

According to the CCK-8, the osteosarcoma cell viability gradually decreased with the increase of CY-09 (NLRP3 inhibitor) concentration and the treatment time (Figure 1D). Colony formation assay revealed that the proliferation ability was suppressed with the increase of the CY-09 concentration (Figure 2A). The transwell assay demonstrated that the migration and invasion abilities of 143B and U2OS cells were also inhibited by increasing the CY-09 concentration (Figure 2B). The suppression of migration ability by CY-09 was also validated by the wound healing assay (Figure 2C).

**FIGURE 3:**
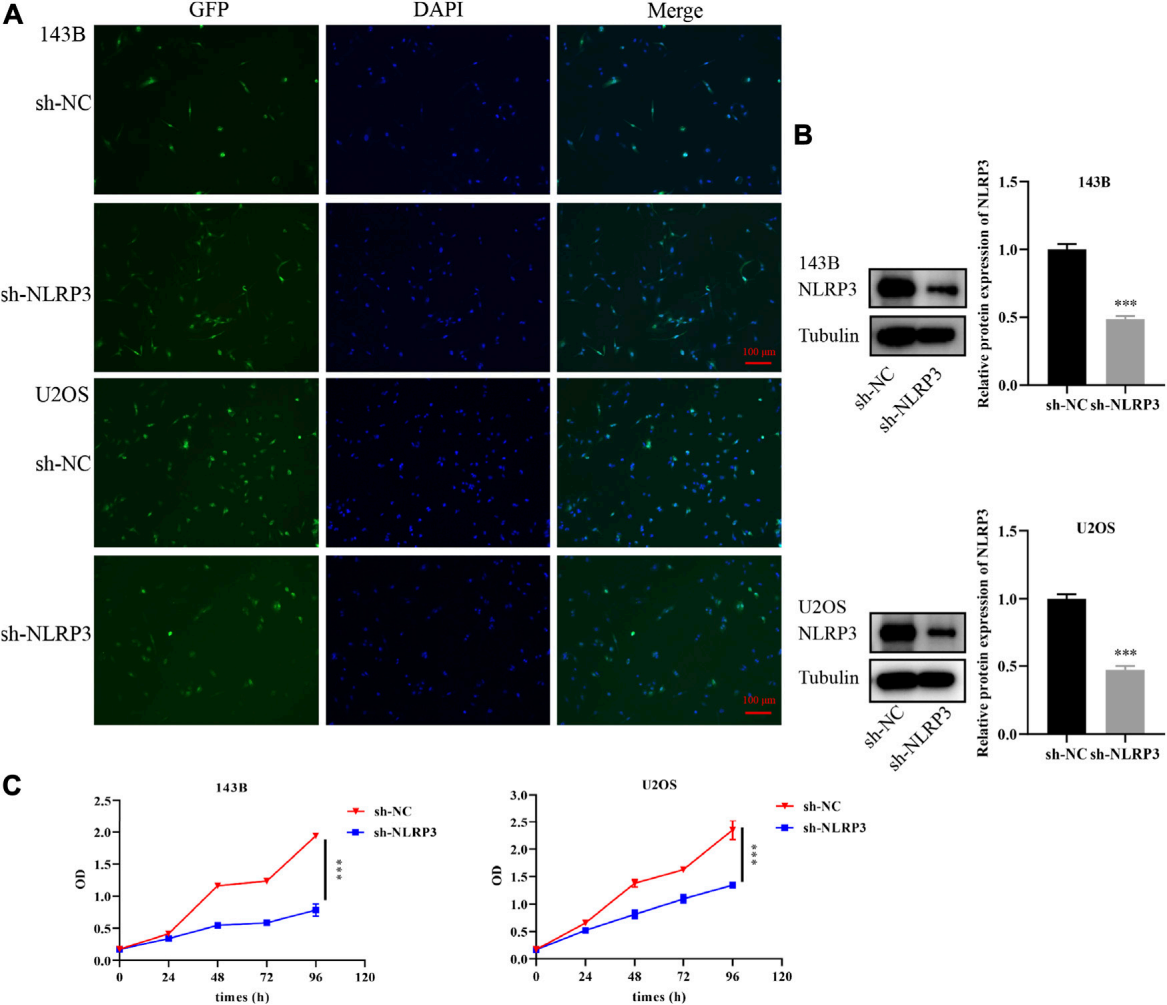
Knockdown of NLRP3 gene and cell viability changes in 143B and U2OS cells **(A)** Infection efficiency of lentivirus in 143B and U2OS cells **(B)** Interference effect of 143B and U2OS cells verified by western blot analysis **(C)** Th changes of cell viability in 143B and U2OS cells after NLRP3 knockdown detected by CCK-8. The experiments were conducted in triplicate.

**FIGURE 4:**
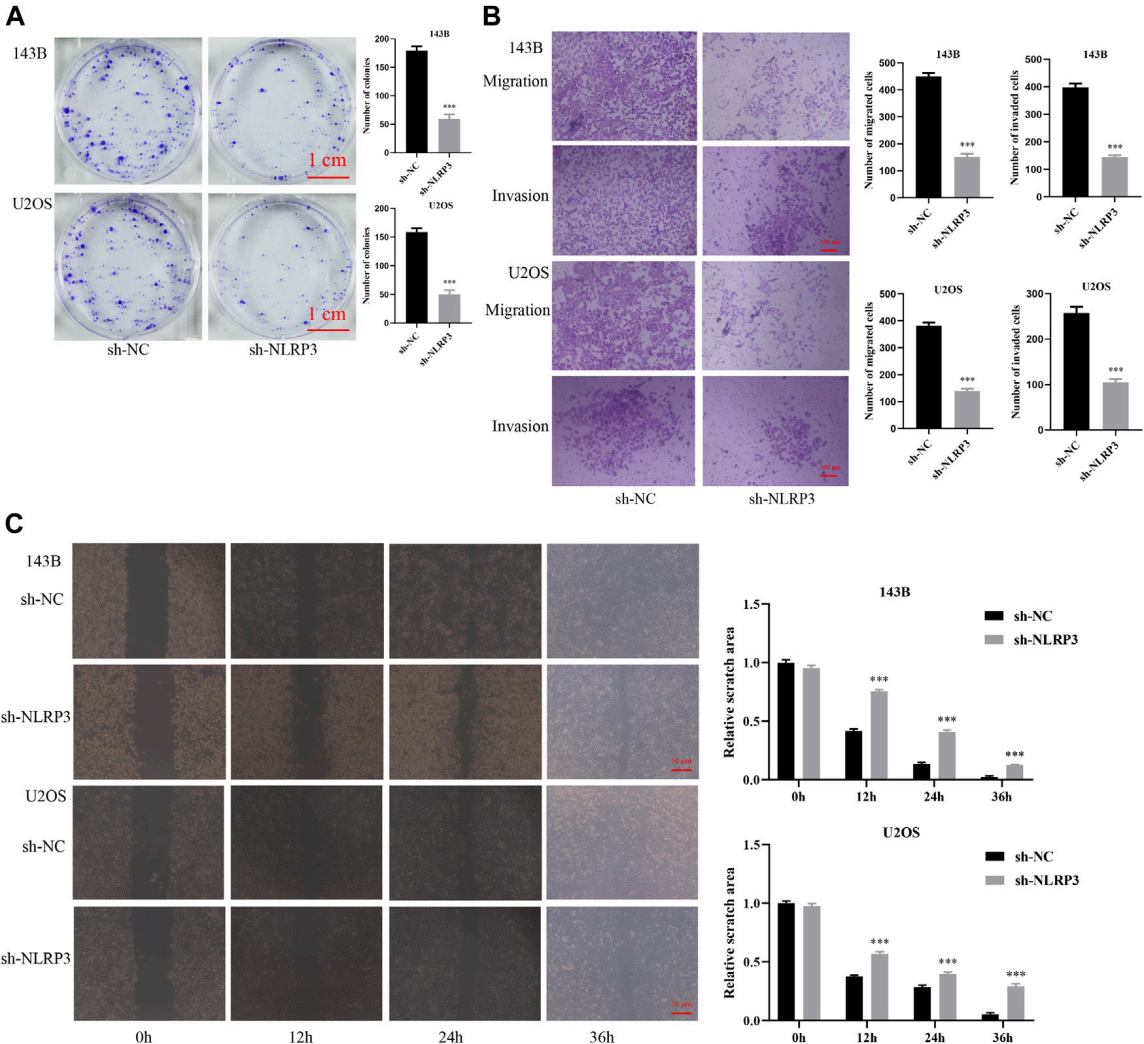
Effect of NLRP3 knockdown on the proliferation, migration, and invasion of 143B and U2OS cells **(A)** Cell proliferation of lentivirus-transfected 143B and U2OS cells detected by colony formation assay after incubation for seven days **(B)** Cell migration and invasion of lentivirus-transfected 143B and U2OS cells examined using the transwell assay **(C)** Cell migration of lentivirus-transfected 143B and U2OS cells detected using the wound healing assay after incubation for 0, 12, 24, and 36 h. The experiments were carried out in triplicate.

To further confirm the target therapy value of NLRP3 for osteosarcoma, lentivirus infection was performed to reduce the expression of the NLRP3 gene in 143B and U2OS cells. Green fluorescence showed the high infection efficiency of lentivirus in 143B and U2OS cells (Figure 3A). Western blot analysis demonstrated decreased NLRP3 protein in 143B and U2OS cells in the NLRP3 knockdown groups (sh-NLRP3) compared to their respective control groups (sh-NC) (Figure 3B). The cell viability was significantly reduced in NLRP3-knockdown 143B and U2OS cells (Figure 3C). Similarly, colony formation assay showed decreased proliferation ability in 143B and U2OS cells after the knockdown of NLRP3 gene (Figure 4A). The transwell assay demonstrated that the migration and invasion abilities was significantly reduced in 143B and U2OS cells undergoing NLRP3 knockdown (Figure 4B). The reduced migration ability of osteosarcoma cells owing to NLRP3 knockdown was also validated via the wound healing assay (Figure 4C).

### NLRP3 Inhibition Induces Cell Apoptosis and G0/G1 Cell Cycle Arrest in Osteosarcoma

**FIGURE 5:**
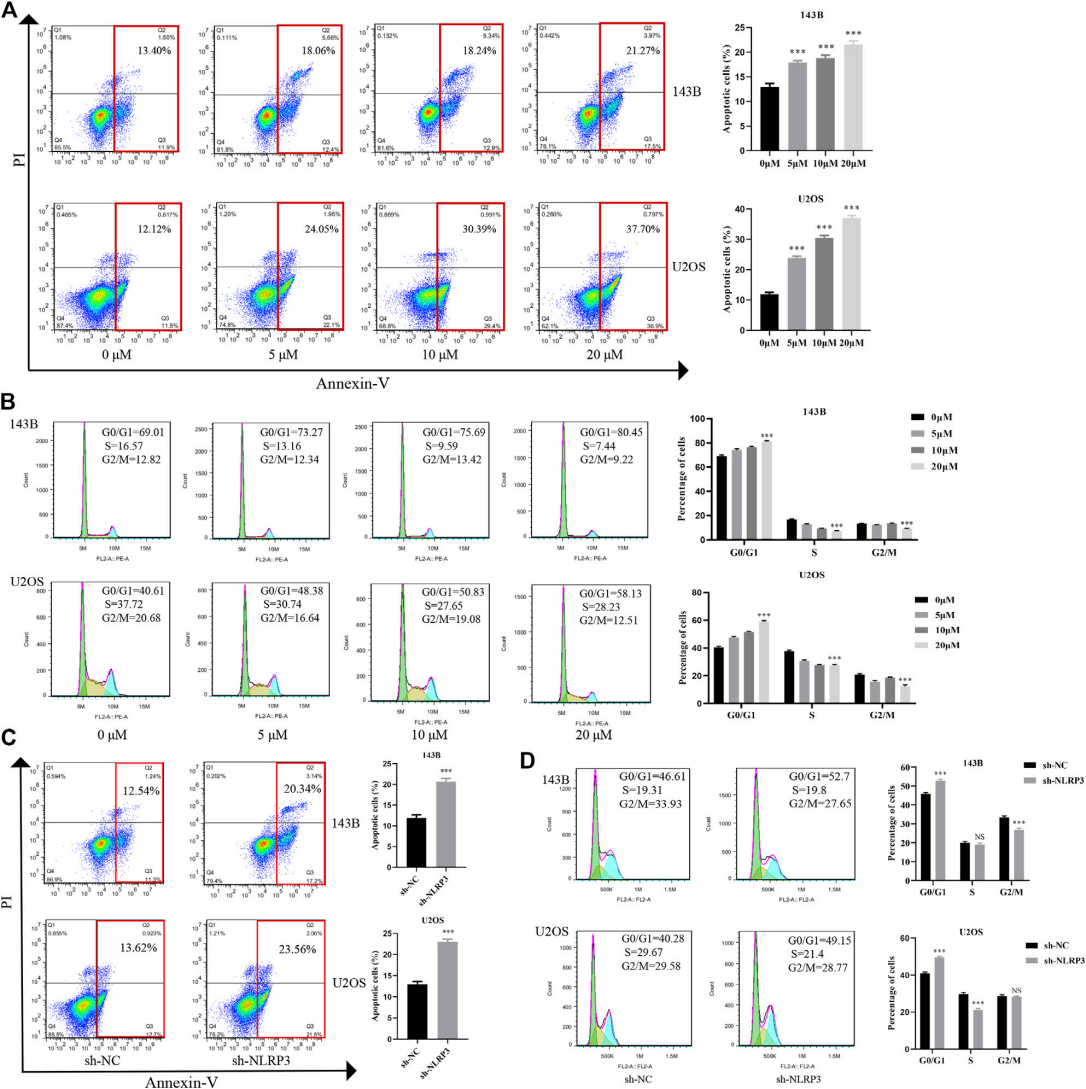
Effect of NLRP3 inhibition on cell apoptosis and cell cycle changes in osteosarcoma **(A)** The cell apoptosis rate of 143B and U2OS cells treated with different concentrations of CY-09 detected using the flow cytometry **(B)** Cell cycle distribution of 143B and U2OS cells treated with different concentrations of CY-09 was detected using the flow cytometry **(C)** The rate of cell apoptosis of lentivirus-transfected 143B and U2OS cells assessed using the flow cytometry **(D)** The cell cycle distribution of lentivirus-transfected 143B and U2OS cells was assessed using the flow cytometry. The experiments were conducted in triplicate.

To examine the role of NLRP3 in cell apoptosis and cell cycle distribution, Annexin V-FITC and PI staining was applied to evaluate the changes of cell apoptosis and cell cycle after CY-09 treatment or the incubation of cells undergoing lentivirus infection for 48 h. The results showed that cell apoptosis increased after the CY-09 intervention and lentivirus knockdown in 143B and U2OS cells via inhibiting cell cycle progression from G0/G1 phase to S and G2/M phases (Figures 5A, B). Similarly, NLRP3 knockdown also induced cell apoptosis and G0/G1 cell cycle arrest in 143B and U2OS cells (Figures 5C, D).

NLRP3 inhibition influences the biological behaviors of osteosarcoma via the NLRP3/Caspaseaspase-1/GSDMD pathway.

**FIGURE 6:**
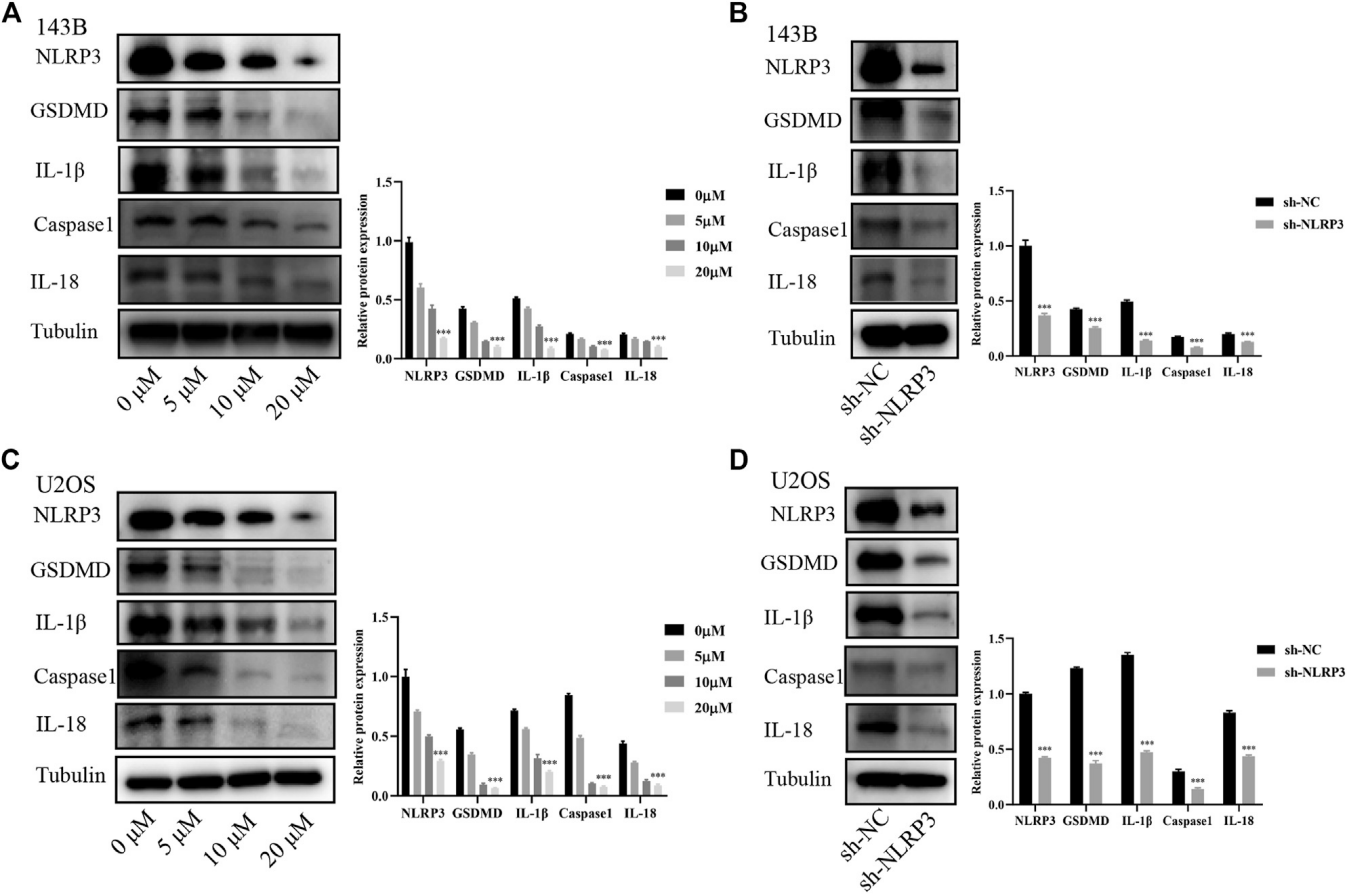
Involvement of the NLRP3/caspase-1/GSDMD pathway in the effect of NLRP3 on osteosarcoma. Expressions of NLRP3/caspase-1/GSDMD and the related proteins IL-1β and IL-18 in 143B cells treated with CY-09 **(A)** or lentivirus infection **(B)** Expressions of NLRP3, caspase-1, GSDMD and the related proteins IL-1β and IL-18 in U2OS cells treated with CY-09 **(C)** or lentivirus infection **(D)** The protein levels were measured in triplicate.

The NLRP3/Caspaseaspase-1/GSDMD pathway has been reported to be crucial for NLRP3 induced tumor progression (Yang et al., 2020). The expression changes of proteins in this signaling pathway were observed in osteosarcoma cells undergoing CY-09 treatment and gene knockdown of NLRP3. Our study found that the expressions of NLRP3 and the downstream proteins including GSDMD, IL-1β, caspase-1 and IL-18 were notably reduced in 143B and U2OS cells after CY-09 treatment or NLRP3 knockdown (Figure 6). These results suggested that NLRP3/Caspase-1/GSDMD pathway may contribute to the effect of NLRP3 on the malignant progression of osteosarcoma.

### NLRP3 Inhibition Regresses the Tumor Growth of Osteosarcoma Cells *in vivo*

**FIGURE 7:**
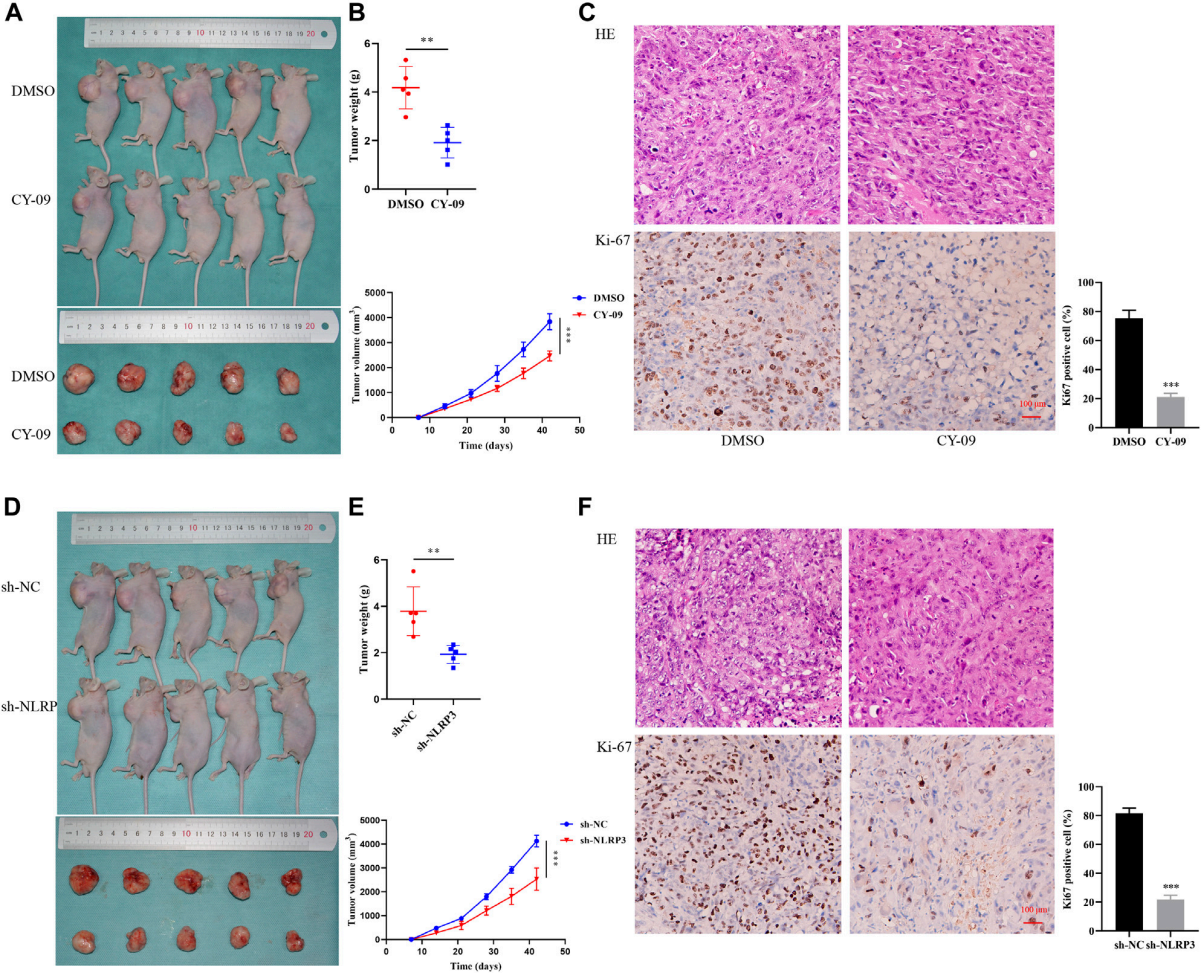
Effect of NLRP3 on tumor formation in osteosarcoma *in vivo* **(A,B)** Images of the xenografted tumor as well as the tumor weight and volume after CY-09 treatment **(C)** H&E staining and immunohistochemical staining of Ki-67 of the osteosarcoma tissues after treatment with CY-09 (D, E) Images of the xenografted tumor as well as the tumor weight and volume after NLRP3 lentivirus infection **(F)** H&E staining, immunohistochemical and statistic staining of Ki-67 of the osteosarcoma tissues after NLRP3 lentivirus infection.

The *in vitro* experiment was species-specific, simpler, more convenient and more detailed, whereas it was at risk of over-interpretation of the results. Therefore, we performed the *in vivo* experiment where the condition recapitulates the reality. We established an osteosarcoma xenograft model using nude mice to evaluate the effects of NLRP3 inhibitor and gene knockdown on tumor formation in osteosarcoma *in vivo*. The results demonstrated that the tumor weight and volume clearly decreased with the CY-09 treatment compared to the control group, which was treated with dimethyl sulfoxide (DMSO) (Figures 7A, B). The weaker expression of Ki-67 of tumors in CY-09 group compared to the DMSO group validated the proliferation inhibition of CY-09 in osteosarcoma (Figure 7C). Similarly, NLRP3 knockdown also reduced the tumor weight and volume of osteosarcoma, as well as the Ki-67 expression in tumor (Figures 7D–F).

## Discussion

Chronic inflammation plays crucial roles in carcinogenesis, tumor progression, and tumor-related inflammation, which have been defined as the seventh hallmark of cancer (Grivennikov et al., 2010; Landskron et al., 2014; Suarez-Carmona et al., 2017). Dysregulation of NLRP3 inflammasome is involved in tumor pathogenesis (Mcallister and Weinberg 2010; Moossavi et al., 2018). However, the real status of NLRP3 in self-antigen modification and upregulation in osteosarcoma carcinogenesis remains unclear. The results obtained in this study observed overexpression of the NLRP3 protein in osteosarcoma tissues, which was closely associated with clinicopathological characteristics including poor response to chemotherapy and distant metastasis. Furthermore, NLRP3 protein was found to be independently related to short PFS and OS in osteosarcoma patients. Suppression of NLRP3 protein by its targeted inhibitor CY-09 or knockdown of the NLRP3 gene could inhibit the cell proliferation, migration, invasion, promote apoptosis and G0/G1 cell cycle arrest in osteosarcoma cells *in vitro via* NLRP3/Caspase-1/GSDMD pathway. Our *in vivo* results further confirmed that the blockage of NLRP3 could suppress the formation of xenografted tumors of osteosarcoma.

Several inflammasomes, including NLRP3, may play a pathogenic role in carcinogenesis through regulating differentiation, adaptive immunity, apoptosis, and gut microbiota (Chen et al., 2020). Some studies have observed significantly high expression of NLRP3 in paraffin-embedded oral squamous cell carcinoma tissues, which was associated with lymph nodes, tumor size, and metastatic status. Moreover, the authors found that overexpression of the NLRP3 protein contributed to the malignant biological behaviors of oral squamous cell carcinoma (Huang et al., 2017; Feng et al., 2018). The present study showed that the NLRP3 protein was overexpressed in osteosarcoma specimens and could be an independent biomarker for the poor clinical outcomes of osteosarcoma patients.

Several types of NLRP3 inhibitors, including those that either directly or indirectly inhibiting the NLRP3 protein, have been reported to date. Directly targeting NLRP3 by small molecules is less invasive whereas more specific and cost-effective than cytokine blockade (Swanson et al., 2019). CY-09 is a selective and direct NLRP3 inhibitor (Jiang et al., 2017). Our results showed that CY-09 suppressed the proliferation, migration, invasion and promoted cell apoptosis of osteosarcoma cells *in vitro*. To investigate the mechanism of NLRP3 blockage on the cell viability, changes of the cell cycle were further examined. The results demonstrated that NLRP3 inhibitor prevented the progression of the cell cycle from G0/G1 to S phases, which may further inhibit the repair process of DNA damage in osteosarcoma cells. Moreover, the *in vivo* results demonstrated that CY-09 regressed the xenografted tumor growth of osteosarcoma. The therapeutic value of CY-09 has been mainly reported in inflammatory diseases, including inflammation after spinal cord injuries Fan et al. (2020) and cerebral ischemia/reperfusion injuries (Sun et al., 2020). Recently, researchers also focused on contributing chronic inflammation created by NLRP3 activation to carcinogenesis, including proliferation, angiogenesis, immunosuppression, and metastasis Liu et al. (2021), Huang et al. (2017), and Chen et al. (2018) showed that blockage of the NLRP3 inflammasome could enhance the antitumor immune responses in head and neck squamous cell carcinoma, suggesting that the NLRP3 inflammasome pathway may be regarded as a novel approach for the treatment of head and neck squamous cell carcinoma. Inhibition of the NLRP3 inflammasome through an NLRP3-specific antagonist could also reduce the cell viability in pancreatic adenocarcinoma (Yaw et al., 2020). Moreover, knockdown of NLRP3 was found to diminish the migration and invasion abilities of colorectal carcinoma cells (Wang et al., 2016). Yin et al. (2018) confirmed that downregulation of NLRP3 could significantly affect glioma cell progression in terms of cellular proliferation, apoptosis, and metastasis. In line with those former studies, our results demonstrated that knockdown of the NLRP3 gene could suppress the malignant progression of osteosarcoma. Therefore, NLRP3 plays a crucial pathogenic role for the tumorigenesis of osteosarcoma.

Activation of the NLRP3 leads to the release of proinflammatory cytokines IL-1β and IL-18 which are mediated by caspase-1, as well as pyroptotic cell death which is mediated by GSDMD (Riestra et al., 2019). Moreover, inhibitor or knockdown of NLRP3 expression can interfere with the formation of the NLRP3 inflammasome, which inhibits the expressions of GSDMD, Caspase-1, IL-1β, and IL-18 protein expression (He et al., 2019; Sanchez-Lopez et al., 2019). The present results of the western blot revealed that the NLRP3 as well as caspase-1, GSDMD, IL-1β, and IL-18 proteins decreased after NLRP3 blockage in osteosarcoma. One previous study by Jin et al. have also observed higher expressions of caspase-1 and its downstream target IL-1β are higher in osteosarcoma cells than in normal cells both *in vitro* and *in vivo* (Jin et al., 2017). Furthermore, a reduced expression of caspase-1 and IL-1β *via* the administration of berberine could significantly suppress the cell viability in osteosarcoma (Jin et al., 2017). Therefore, NLRP3/Caspase-1/GSDMD pathway may be involved in NLRP3-induced carcinogenesis of osteosarcoma.

There were some limitations to this study. Firstly, selection bias should be considered due to the retrospective nature of the study. In addition, the HR with wide CI may be inflated due to monotone likelihood phenomenon (Tzeng 2021). Further large-scale studies with a longer follow-up time should be performed for more convincing evidence.

To sum up, our results showed that overexpression of the NLRP3 protein was closely associated with the adverse clinicopathological characteristics of osteosarcoma. Additionally, NLRP3 may be regarded as an independent prognostic biomarker for osteosarcoma. The inhibition of NLRP3 by CY-09 or gene knockdown significantly suppressed the cell proliferation, tumor formation, migration, invasion, induced cell apoptosis and G0/G1 cell cycle arrest in osteosarcoma. Individualized therapy targeting NLRP3 may serve as a modality to prevent tumor progression of osteosarcoma. The findings obtained in this study may be promising for osteosarcoma patients because of the limitations of current therapies and relative stagnation of patient outcomes over the past decades. Nevertheless, further investigations are required to confirm the therapeutic value of NLRP3 in osteosarcoma.

## Data Availability

The raw data supporting the conclusions of this article will be made available by the authors, without undue reservation.

## References

[B1] Chen L. Huang C.-F. Li Y.-C. Deng W.-W. Mao L. Wu L. (2018). Blockage of the NLRP3 Inflammasome by MCC950 Improves Anti-tumor Immune Responses in Head and Neck Squamous Cell Carcinoma. Cell. Mol. Life Sci. 75 (11), 2045–2058. 10.1007/s00018-017-2720-9 29184980 PMC11105265

[B2] Chen Y. Yang Q. Lv C. Chen Y. Zhao W. Li W. (2020). NLRP3 Regulates Alveolar Bone Loss in Ligature-Induced Periodontitis by Promoting Osteoclastic Differentiation. Cell Prolif 54, e12973. 10.1111/cpr.12973 33382502 PMC7849172

[B3] Chow T. Wutami I. Lucarelli E. Choong P. F. Duchi S. Di Bella C. (2020). Creating *In Vitro* Three-Dimensional Tumor Models: A Guide for the Biofabrication of a Primary Osteosarcoma Model. Tissue Eng. Part. B Rev. 10.1089/ten.teb.2020.0254 33138724

[B4] Dai X. Ma W. He X. Jha R. K. (2011). Review of Therapeutic Strategies for Osteosarcoma, Chondrosarcoma, and Ewing's Sarcoma. Med. Sci. Monit. 17 (8), RA177–RA190. 10.12659/msm.881893 21804475 PMC3539609

[B5] Fan X. Ma W. Zhang Y. Zhang L. (2020). P2X7 Receptor (P2X7R) of Microglia Mediates Neuroinflammation by Regulating (NOD)-Like Receptor Protein 3 (NLRP3) Inflammasome-dependent Inflammation after Spinal Cord. Inj. Med Sci Monit 26, e925491. 10.12659/msm.925491 PMC7518010 32952148

[B6] Feng X. Luo Q. Wang H. Zhang H. Chen F. (2018). MicroRNA‐22 Suppresses Cell Proliferation, Migration and Invasion in Oral Squamous Cell Carcinoma by Targeting NLRP3. J. Cel Physiol 233 (9), 6705–6713. 10.1002/jcp.26331 PMC13482448 29319163

[B7] Grivennikov S. I. Greten F. R. Karin M. (2010). Immunity, Inflammation, and Cancer. Cell 140 (6), 883–899. 10.1016/j.cell.2010.01.025 20303878 PMC2866629

[B8] He W. Long T. Pan Q. Zhang Y. Zhang S. Zhang D. (2019). Microglial NLRP3 Inflammasome Activation Mediates IL-1beta Release and Contributes to central Sensitization in a Recurrent Nitroglycerin-Induced Migraine Model. J. Neuroinflammation 16 (1), 78. 10.1186/s12974-019-1459-7 30971286 PMC6456991

[B9] Huang C. F. Chen L. Li Y. C. Wu L. Yu G. T. Zhang W. F. (2017). NLRP3 Inflammasome Activation Promotes Inflammation-Induced Carcinogenesis in Head and Neck Squamous Cell Carcinoma. J. Exp. Clin. Cancer Res. 36 (1), 116. 10.1186/s13046-017-0589-y 28865486 PMC5581464

[B10] Jiang H. He H. Chen Y. Huang W. Cheng J. Ye J. (2017). Identification of a Selective and Direct NLRP3 Inhibitor to Treat Inflammatory Disorders. J. Exp. Med. 214 (11), 3219–3238. 10.1084/jem.20171419 29021150 PMC5679172

[B11] Jin H. Jin X. Cao B. Wang W. (2017). Berberine Affects Osteosarcoma via Downregulating the caspase-1/IL-1β Signaling axis. Oncol. Rep. 37 (2), 729–736. 10.3892/or.2016.5327 28000894 PMC5355653

[B12] Landskron G. De la Fuente M. Thuwajit P. Thuwajit C. Hermoso M. A. (2014). Chronic Inflammation and Cytokines in the Tumor Microenvironment. J. Immunol. Res. 2014, 149185. 10.1155/2014/149185 24901008 PMC4036716

[B13] Li A. Y. Atenafu E. G. Bernard R. S. Masih-Khan E. Reece D. Franke N. (2020). Toxicity and Survival Outcomes of Autologous Stem Cell Transplant in Multiple Myeloma Patients with Renal Insufficiency: an Institutional Comparison between Two Eras. Bone Marrow Transpl. 55 (3), 578–585. 10.1038/s41409-019-0697-8 31558786

[B14] Lin Y. Luo T. Weng A. Huang X. Yao Y. Fu Z. (2020). Gallic Acid Alleviates Gouty Arthritis by Inhibiting NLRP3 Inflammasome Activation and Pyroptosis through Enhancing Nrf2 Signaling. Front. Immunol. 11, 580593. 10.3389/fimmu.2020.580593 33365024 PMC7750458

[B15] Liu C.-C. Miao Y. Chen R.-L. Zhang Y.-Q. Wu H. Yang S.-M. (2021). STIM1 Mediates IAV-Induced Inflammation of Lung Epithelial Cells by Regulating NLRP3 and Inflammasome Activation via Targeting miR-223. Life Sci. 266, 118845. 10.1016/j.lfs.2020.118845 33278394

[B16] Liu S.-G. Wu X.-X. Hua T. Xin X.-y. Feng D.-L. Chi S.-Q. (2019). NLRP3 Inflammasome Activation by Estrogen Promotes the Progression of Human Endometrial Cancer. Ott 12, 6927–6936. 10.2147/ott.s218240 PMC6717726 31695408

[B17] McAllister S. S. Weinberg R. A. (2010). Tumor-host Interactions: a Far-Reaching Relationship. Jco 28 (26), 4022–4028. 10.1200/jco.2010.28.4257 20644094

[B18] Moossavi M. Parsamanesh N. Bahrami A. Atkin S. L. Sahebkar A. (2018). Role of the NLRP3 Inflammasome in Cancer. Mol. Cancer 17 (1), 158. 10.1186/s12943-018-0900-3 30447690 PMC6240225

[B19] Riestra A. M. Valderrama J. A. Patras K. A. Booth S. D. Quek X. Y. Tsai C.-M. (2019). Trichomonas Vaginalis Induces NLRP3 Inflammasome Activation and Pyroptotic Cell Death in Human Macrophages. J. Innate Immun. 11 (1), 86–98. 10.1159/000493585 30391945 PMC6296884

[B20] Sanchez-Lopez E. Zhong Z. A. Stubelius. Stubelius A. Sweeney S. R. Booshehri L. M. Antonucci L. (2019). Choline Uptake and Metabolism Modulate Macrophage IL-1β and IL-18 Production. Cel Metab. 29 (6), 1350–1362. 10.1016/j.cmet.2019.03.011 PMC6675591 30982734

[B21] Schisterman E. F. Perkins N. J. Liu A. Bondell H. (2005). Optimal Cut-point and its Corresponding Youden Index to Discriminate Individuals Using Pooled Blood Samples. Epidemiology 16 (1), 73–81. 10.1097/01.ede.0000147512.81966.ba 15613948

[B22] Srirajaskanthan R. Shah T. Watkins J. Marelli L. Khan K. Caplin M. E. (2010). Expression of the HER-1-4 Family of Receptor Tyrosine Kinases in Neuroendocrine Tumours. Oncol. Rep. 23 (4), 909–915. 10.3892/or_00000714 20204273

[B23] Suarez-Carmona M. Lesage J. Cataldo D. Gilles C. (2017). EMT and Inflammation: Inseparable Actors of Cancer Progression. Mol. Oncol. 11 (7), 805–823. 10.1002/1878-0261.12095 28599100 PMC5496491

[B24] Sun R. Peng M. Xu P. Huang F. Xie Y. Li J. (2020). Low-density Lipoprotein Receptor (LDLR) Regulates NLRP3-Mediated Neuronal Pyroptosis Following Cerebral Ischemia/reperfusion Injury. J. Neuroinflammation 17 (1), 330. 10.1186/s12974-020-01988-x 33153475 PMC7643474

[B25] Swanson K. V. Deng M. Ting J. P.-Y. (2019). The NLRP3 Inflammasome: Molecular Activation and Regulation to Therapeutics. Nat. Rev. Immunol. 19 (8), 477–489. 10.1038/s41577-019-0165-0 31036962 PMC7807242

[B26] Tzeng I. S. (2021). To Handle the Inflation of Odds Ratios in a Retrospective Study with a Profile Penalized Log-Likelihood Approach. J. Clin. Lab. Anal. 35, e23849. 10.1002/jcla.23849 34043251 PMC8274998

[B27] Wang H. Luo Q. Feng X. Zhang R. Li J. Chen F. (2018). NLRP3 Promotes Tumor Growth and Metastasis in Human Oral Squamous Cell Carcinoma. BMC Cancer 18 (1), 500. 10.1186/s12885-018-4403-9 29716544 PMC5930757

[B28] Wang H. Wang Y. Du Q. Lu P. Fan H. Lu J. (2016). Inflammasome-independent NLRP3 Is Required for Epithelial-Mesenchymal Transition in colon Cancer Cells. Exp. Cel Res. 342 (2), 184–192. 10.1016/j.yexcr.2016.03.009 26968633

[B29] Wang S. L. Zhong G. X. Wang X. W. Yu F. Q. Weng D. F. Wang X. X. (2018). Prognostic Significance of the Expression of HER Family Members in Primary Osteosarcoma. Oncol. Lett. 16 (2), 2185–2194. 10.3892/ol.2018.8931 30008917 PMC6036504

[B30] Xue Y. Du H. D. Tang D. Zhang D. Zhou J. Zhai C. W. (2019). Correlation between the NLRP3 Inflammasome and the Prognosis of Patients with LSCC. Front. Oncol. 9, 588. 10.3389/fonc.2019.00588 31312615 PMC6614490

[B31] Yang Y. Liu P. Y. Bao W. Chen S. J. Wu F. S. Zhu P. Y. (2020). Hydrogen Inhibits Endometrial Cancer Growth via a ROS/NLRP3/caspase-1/GSDMD-mediated Pyroptotic Pathway. BMC Cancer 20 (1), 28. 10.1186/s12885-019-6491-6 31924176 PMC6954594

[B32] Yang Y. Wang H. Kouadir M. Song H. Shi F. (2019). Recent Advances in the Mechanisms of NLRP3 Inflammasome Activation and its Inhibitors. Cell Death Dis 10 (2), 128. 10.1038/s41419-019-1413-8 30755589 PMC6372664

[B33] Yaw A. C. K. Chan E. W. L. Yap J. K. Y. Mai C. W. (2020). The Effects of NLRP3 Inflammasome Inhibition by MCC950 on LPS-Induced Pancreatic Adenocarcinoma Inflammation. J. Cancer Res. Clin. Oncol. 146 (9), 2219–2229. 10.1007/s00432-020-03274-y 32507974 PMC11804369

[B34] Yin X. F. Zhang Q. Chen Z. Y. Wang H. F. Li X. Wang H. X. (2018). NLRP3 in Human Glioma Is Correlated with Increased WHO Grade, and Regulates Cellular Proliferation, Apoptosis and Metastasis via Epithelial-Mesenchymal Transition and the PTEN/AKT Signaling Pathway. Int. J. Oncol. 53 (3), 973–986. 10.3892/ijo.2018.4480 30015880 PMC6065456

