# Corrigendum: NLRP3 Overexpression Associated With Poor Prognosis and Presented as an Effective Therapeutic Target in Osteosarcoma

**DOI:** 10.3389/fphar.2021.753231

**Published:** 2021-09-02

**Authors:** Zhen Huang, Hui Chen, Shenglin Wang, Hongxiang Wei, Xinwen Wang, Rongkai Shen, Yunqing Wang, Rongjin Lin, Jianhua Lin

**Affiliations:** ^1^ Department of Rehabilitation, The First Affiliated Hospital of Fujian Medical University, Fuzhou, China; ^2^ Department of Orthopedics, The First Affiliated Hospital of Fujian Medical University, Fuzhou, China; ^3^ Fujian Orthopedics Research Institution, The First Affiliated Hospital of Fujian Medical University, Fuzhou, China; ^4^ Department of Nephrology, Shanghai East Hospital, School of Medicine, Tongji University, Shanghai, China; ^5^ Department of Orthopedics, The People’s Hospital of Jiangmen City, Southern Medical University, Jiangmen, China; ^6^ Department of Nursing, The First Affiliated Hospital of Fujian Medical University, Fuzhou, China

**Keywords:** osteosarcoma, prognosis, NLRP3, CY-09, lentivirus

In the original article, there was a mistake in Figure 3 as published. In the process of rearranging our published articles, we found a problem by accident that the tubulin blots in Figure 3B were mistakenly uploaded. The corrected Figure 3 appears below.

**FIGURE 3:**
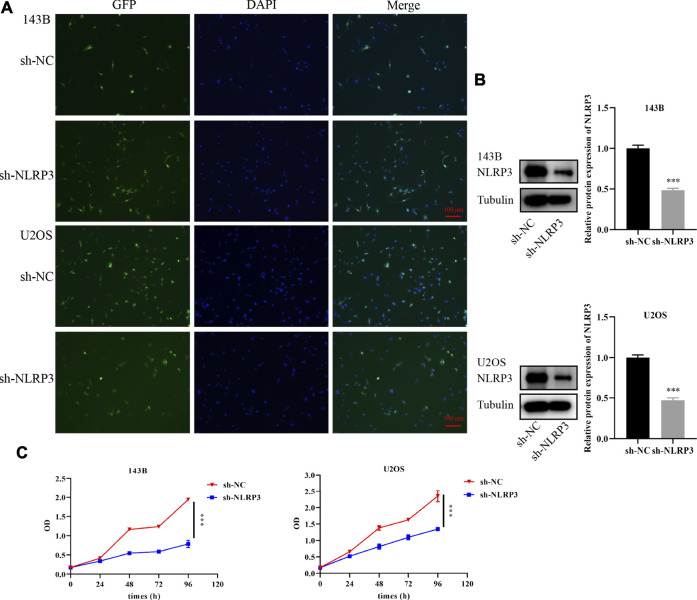
Knockdown of NLRP3 gene and cell viability changes in 143B and U2OS cells **(A)** Infection efficiency of lentivirus in 143B and U2OS cells **(B)** Interference effect of 143B and U2OS cells verified by western blot analysis **(C)** Th changes of cell viability in 143B and U2OS cells after NLRP3 knockdown detected by CCK-8. The experiments were conducted in triplicate.

The authors apologize for this error and state that this does not change the scientific conclusions of the article in any way. The original article has been updated.

